# Individual units rather than entire hospital as the basis for improvement: the example of two Methicillin resistant Staphylococcus aureus cohort studies

**DOI:** 10.1186/2047-2994-1-8

**Published:** 2012-02-13

**Authors:** Petra Gastmeier, Frank Schwab, Iris Chaberny, Christine Geffers

**Affiliations:** 1 Institute of Hygiene and Environmental Medicine Charité-University Hospital Berlin, Hindenburgdamm 27, 12203 Berlin, Germany; 2 Institute of Medical Microbiology and Hospital Epidemiology Hannover Medical School, Carl-Neuberg-Str, 1 30625, Hannover Berlin, Germany

**Keywords:** Infection prevention, Surveillance, MRSA, Quality management

## Abstract

**Background:**

Two MRSA surveillance components exist within the German national nosocomial infection surveillance system KISS: one for the whole hospital (i.e. only hospital based data and no rates for individual units) and one for ICU-based data (rates for each individual ICU). The objective of this study was to analyze which surveillance system (a hospital based or a unit based) leads to a greater decrease in incidence density of nosocomial MRSA

**Methods:**

Two cohort studies of surveillance data were used: Data from a total of 224 hospitals and 359 ICUs in the period from 2004 to 2009. Development over time was described first for both surveillance systems. In a second step only data were analyzed from those hospitals/ICUs with continuous participation for at least four years. Incidence rate ratios (IRR) with 95% confidence intervals were calculated to compare incidence densities between different time intervals.

**Results:**

In the baseline year the mean MRSA incidence density of hospital acquired MRSA cases was 0.25 and the mean incidence density of ICU-acquired MRSA was 1.25 per 1000 patient days. No decrease in hospital-acquired MRSA rates was found in a total of 111 hospitals with continuous participation in the hospital- based system. However, in 159 ICUs with continuous participation in the unit-based system, a significant decrease of 29% in ICU-acquired MRSA was identified.

**Conclusions:**

A unit-based approach of surveillance and feedback seems to be more successful in decreasing nosocomial MRSA rates, compared to a hospital-based approach. Therefore each surveillance system should provide unit-based data to stimulate activities on the unit level.

## Background

Aspects of hospital infection control have achieved a great deal of interest from the media, the public and politicians in many countries and many hospitals worldwide are very active in improving the situation. Meanwhile, a considerable body of knowledge about the effectiveness and suitability of specific infection control measures has been established. Comprehensive guidelines have been developed in many countries to support the infection control recommendations in individual hospitals. However, knowledge about the best infection control measures is often not the most important point in improving the situation. In many hospitals compliance with these recommendations is far from ideal and barriers for low compliance must be identified to overcome them.

One of the most interesting infection control problems is the MRSA (Methicillin resistant Staphylococcus aureus) problem. Many hospitals have introduced surveillance systems to monitor MRSA import and the development of nosocomial MRSA cases. National surveillance systems support them by providing definitions, protocols and reference data.

The German national nosocomial infection surveillance system (KISS) has established a hospital-wide surveillance system in 2003 (pilot year) which is called MRSA-KISS to track MRSA cases on the hospital level [1]. The hospitals provide data about imported and hospital-acquired MRSA cases once a year, and the denominator data are the number of patient-days of the whole hospital. Reference data are stratified by hospital size and screening frequency and published once a year.

Further extensive surveillance of MRSA cases is performed on the unit level within the ICU surveillance component. The ICUs have been required since 2003 to provide information about all ICU-acquired and imported cases (infections and colonizations) of multidrug resistant organisms (multidrug resistant organisms (MDRO), such as MRSA, VRE, ESBL) in addition to and independently from surveillance of lower respiratory tract infections, primary bloodstream infections, urinary tract infections and meningitis/ventriculits [2,3]. During ICU-MDRO surveillance the units enter this information monthly on a unit-by-unit basis in a web based surveillance system and are able to generate analyses of their data at any time.

Meanwhile we have an overview about more than 6 years in both MRSA surveillance systems. The objective of this study therefore was to analyze which surveillance system (hospital-based or unit- based) leads to a greater decrease in incidence density of newly acquired MRSA cases.

## Method

Two cohort studies of surveillance data were conducted. Data from a total of 224 hospitals providing hospital based nosocomial MRSA incidence rates and 359 ICUs providing unit based nosocomial MRSA incidence density rates analyzed with data from the years 2004 to 2009. Data from 2003 were excluded because this year was a pilot phase for both surveillance systems.

Annual incidence density was calculated (i.e., the number of MRSA cases standardized per 1000 patient days) to describe the change of MRSA cases over the years. MRSA cases in both surveillance systems were considered imported when MRSA reports from prior admissions or discharge reports or microbiological results from surveillance cultures or clinical specimens taken within the first 48 hours following admission were available. The screening frequency was defined as the number of nasal swabs taken per 1000 patient days, whereby only the first swab per patient was counted. The term "nosocomial was applied to hospital acquired cases in MRSA-KISS (case is acquired for the hospital following admission to that hospital) and to ICU-acquired cases (a case is acquired for an ICU following admission to that ICU) from data of the MDRO component of ICU KISS.

The development of incidence density over time (calendar years) was described first for both surveillance components. All participating hospitals or ICUs were included. Because a surveillance effect can be expected after at least four years of surveillance, the data from those hospitals/ICUs with continuous participation for at least four years were analyzed in a second step. Only the data from the first four years of participation were included in the analysis.

Incidence rate ratios (IRR) with 95% confidence intervals were calculated to compare incidence densities for hospital- and ICU-acquired cases between the different time intervals. SAS 9.2 (SAS Institute Inc., Cary, NC, USA) PASW Statistics 18 and EpiInfo version 6.04 were used for analysis.

## Results

### Hospital-wide MRSA surveillance

**Figure 1:**
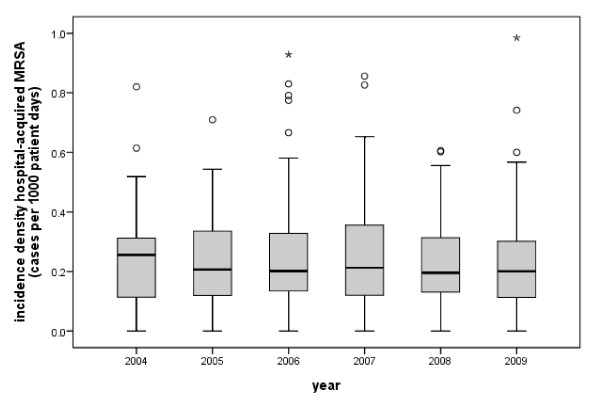
**Incidence density of hospital acquired MRSA cases stratified by year**.

A total of 224 hospitals provided their MRSA data during the period from 2004 to 2009 with an increasing number of hospitals participating from year to year (Table 1) and a huge variety in rates among the hospitals (Figure 1). The overall MRSA incidence density was 0.94 per 1000 patient days, and the incidence density of hospital-acquired cases was 0.25 per 1000 patient days. From 2004 to 2007, the incidence density of hospital-acquired cases increased by 7%. Since 2007 the incidence density of hospital acquired cases has decreased by 14%.

**Table 1:** Data from 224 hospitals participating in MRSA-KISS from 2004 to 2009

Year	Hospitals	MRSA cases			Patient days	MRSA incidence density/per 1000 patient days			IRR	
		**Total**	**Imported (%)**	**Hospital acquired**		**Total**	**Im-ported**	**Hospital acquired**	**Hospital acquired**	**CI95**

2004	63	5651	3327 (59)	2324	9421634	0,60	0,35	0,25	1 = reference	

2005	88	9124	5885 (65)	3239	12808024	0,71	0,46	0,25	1.03	0.97-1.08

2006	115	13810	9631 (70)	4179	15957877	0,87	0,60	0,26	1.06	1.01-1.12

2007	148	19147	13991 (73)	5156	19594818	0,98	0,71	0,26	1.07	1.02-1.12

2008	178	24190	18493 (76)	5697	23864511	1,01	0,77	0,24	0.97	0.92-1.02

2009	204	28628	22822 (80)	5806	25416849	1,13	0,90	0,23	0.93	0.88-0.97

Total	224	100550	74149 (74)	26401	107063713	0,94	0,69	0,25		

Table 2 shows the development over the first four years of participation in 111 hospitals with continuous MRSA-KISS participation for at least four years. The total incidence density of MRSA increased, but the incidence density of hospital acquired MRSA did not show any changes over time. Hospitals with continuous MRSA-KISS participation had a lower overall MRSA incidence density compared to those with shorter periods of participation, but the incidence density of hospital acquired cases was the same.

**Table 2:** Data from 111 hospitals with continuous participation in MRSA-KISS for at least four years

Year of partici-pation	Hospitals	MRSA cases			Patient days	MRSA incidence density/per 1000 patient days			IRR Hospital acquired	CI95
		**Total**	**Imported (%)**	**Hospital acquired**		**Total**	**Im-ported**	**Hospital acquired**		

1.	111	10615	6949 (65)	3666	14830273	0,72	0,47	0,25	1 = reference	-

2.	111	13057	9140 (70)	3917	15220824	0,86	0,60	0,26	1.04	1.00-1.09

3.	111	14723	10840 (74)	3883	15044052	0,98	0,72	0,26	1.04	1.00-1.09

4.	111	16321	12368 (76)	3953	15441101	1,06	0,80	0,26	1.04	0.99-1.08

Total	111	54716	39297 (72)	15419	60536250	0,90	0,65	0,25	-	-

During the observation period the frequency of MRSA screening in most of the hospitals participating in MRSA-KISS increased, but not all hospitals were able to provide information about their screening frequency. During the first year of participation, 56 of 111 hospitals were able to provide this information, and the median frequency was 1.6 patients with at least one nasal swab per 1000 patient days. During the fourth year 74 from 111 hospitals were able to provide screening frequencies, and the median was 4.3 per 1000 patient days.

### ICU-wide MRSA surveillance data

**Figure 2:**
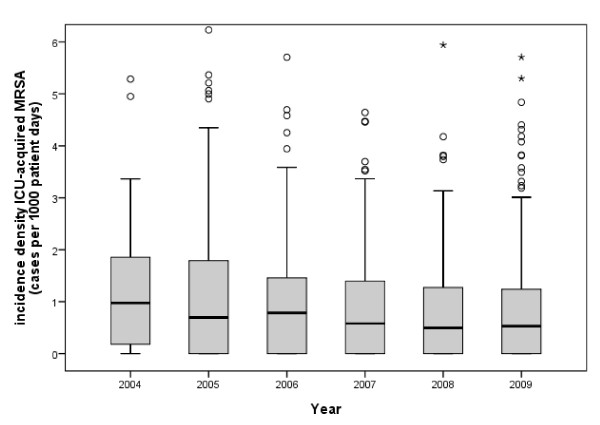
**Incidence density of ICU acquired MRSA cases**.

A total of 359 ICUs provided their MRSA data during the period from 2004 to 2009 with an increasing number of participating ICUs from year to year (Table 3) and also with substantial variation among ICUs (Figure 2). The overall MRSA incidence density was 3.99 per 1000 patient days. That means it was about four times higher than at the hospital level. The incidence density of ICU acquired cases was 1.00 per 1000 patient days. In other words, a quarter of all MRSA cases were ICU-acquired. This holds true for hospital-acquired MRSA as well (see Table 1). From 2004 to 2008 a decrease of ICU-acquired MRSA was observed with no further decrease in 2009.

**Table 3:** Data from 359 ICU participating in the MDRO component of ICU-KISS from 2004 to 2009

Year	ICUs	MRSA cases			Patient days	MRSA incidence density/per 1000 patient days			RR	
		**Total**	**Imported to ICU (%)**	**ICU-acquired**		**total**	**Im-ported to ICU**	**ICU- acquired**	**ICU- acquired**	**CI95**

2004	93	1085	702 (65)	383	287141	3.78	2.44	1.33	1 = reference	-

2005	175	2162	1492 (69)	670	526502	4.11	2.83	1.27	0.95	0.84-1.08

2006	201	2681	1978 (74)	703	623286	4.30	3.17	1.13	0.85	0.75-0.96

2007	247	2915	2229 (76)	686	727799	4.01	3.06	0.94	0.71	0.62-0.80

2008	257	3035	2392 (79)	643	784580	3.87	3.05	0.82	0.61	0.54-0.70

2009	291	3481	2713 (78)	768	903291	3.85	3.00	0.85	0.64	0.56-0.72

Total	359	15359	11506 (75)	3853	3852599	3.99	2.99	1.00		

Table 4 shows the development over the first four years of participation in 159 ICUs with continuous participation in ICU-MDRO-KISS for at least four years. The incidence density of ICU acquired MRSA decreased significantly by 29% over the four year period in these ICUs. The ICUs with continuous participation are not considerably different from the total group of ICUs providing data in the period from 2004-2009.

**Table 4:** Data from 159 ICUs with continuous participation in ICU-KISS over at least four years

Year of partici-pation	ICUs	MRSA cases			Patient days	MRSA incidence density/per 1000 patient days			IRR ICU-acquired	CI95
		**Total**	**Imported to ICU (%)**	**ICU- acquired**		**Total**	**Im-ported to ICU**	**ICU- acquired**		

1.	159	2033	1358 (67)	675	538363	3.78	2.52	1.25	1 = reference	-

2.	159	2166	1595 (74)	571	543310	3.99	2.94	1.05	0.84	0.75-0.94

3.	159	2232	1697 (76)	535	537358	4.15	3.16	1.00	0.79	0.71-0.89

4.	159	2097	1635 (78)	462	520039	4.03	3.14	0.89	0.71	0.63-0.80

Total	159	8528	6285 (74)	2243	2139070	3.99	2.94	1.05		-

In 42 hospitals with continuous MRSA KISS participation their ICUs also continuously took part in the MDRO component of ICU KISS. For this subgroup of hospitals we also investigated the development according to the year of participation. An even higher reduction was found over the four year period (IRR = 0.66) in these ICUs, whereas in the remaining hospitals (without the ICUs) no significant changes were found (IRR = 0.97).

## Discussion

National guidelines for MRSA prevention were published in Germany in 1999 [4] and each hospital must record the occurrence of MDRO such as MRSA since 2001, but without mandatory reporting [5]. German microbiology laboratories have been participating in the European Antimicrobial Resistance Surveillance System (EARSS) and have provided the MRSA percentage of *S.aureus *bacteremia isolates for many years. Since 2008 a more comprehensive surveillance system for MDRO from laboratory data has been established (ARS = Antibiotika Resistenz-Surveillance, https://ars.rki.de). In 2009 a mandatory reporting of MRSA bacteremia cases was introduced [6]. MRSA-KISS and the MDRO component of ICU-KISS were established in 2003 as voluntary surveillance systems. In 2009 about 10% of all hospitals were participating and about 15% of all ICUs.

Compared to the data from other countries the incidence density of nosocomial MRSA in German hospitals and ICUs is lower (especially considering that most publications report only nosocomial MRSA infections, not nosocomial MRSA cases (i.e. including colonized as well as infected patients) [7-9]. But of course there are also countries with lower MRSA rates. One example is the report from 38 French hospitals describing a decrease of the MRSA incidence density from 1.16 to 0.57 per 1000 hospital days between 1993 and 2007 [10].

Intensive care units participating in the MDRO component of ICU-KISS were able to achieve a significant decrease of ICU-acquired MRSA cases of 29% during a four-year surveillance period. This is in accordance with similar observations concerning the influence of surveillance activities on the development of nosocomial pneumonia and primary bloodstream infections in ICU-KISS. Significant reductions of infection rates of between 14 and 29% were demonstrated during various periods of analysis [11-14]. On the unit level, the surveillance staff together with the ICU staff can analyze the data at any time, present the information to the ICU staff and stimulate discussions to analyze reasons for infection control problems and to introduce the most appropriate interventions. Already in the second year of surveillance a significant effect was observed with further improvement in the following years.

However, no decrease in hospital-acquired MRSA rates on the hospital level was found. Normally, MRSA rates on the hospital level are presented during meetings of the hospital infection control committees. But, even if the hospitals have hospital-acquired MRSA rates above the median, the hospital committees are often not able to identify reasons for this situation and draw the most appropriate conclusions. A similar development was demonstrated within the other hospital-based surveillance system in KISS, the surveillance system for Clostridium difficile associated diseases (CDAD-KISS), where we also did not find a reduction of nosocomial cases within the first 3 years of participation. Perhaps the infection control staff has focused its activities on the ICU within their hospitals because they know that the highest MRSA rates can be observed on the ICUs and they are used to work in the field of quality management with the ICUs due to the experience from ICU KISS.

Moreover, many hospitals increased their admission screening frequency remarkably during the observation period. Without admission screening, cases with an MRSA positive microbiology report from day 3 on will be automatically considered hospital-acquired or ICU-acquired according to the MRSA-KISS and ICU-MDRO protocols. With admission screening, on the other hand, many cases will be classified as imported which would not be the case without admission screening. Due to this reclassification the constant hospital-acquired MRSA incidence in MRSA-KISS hospitals with continuous participation will be in reality associated with an increase of hospitals-acquired cases. For the ICUs we do not have the data about the development of admission screening. But we also expect an increase over the years along with the increase in the entire hospitals. Therefore the observed 29% decrease of the ICU-acquired MRSA incidence density has also to be regarded-at least in part-in the light of this re-classification bias.

Unfortunately we do not have comparable data from hospitals without continuous MRSA surveillance in Germany. The only data source available is the above mentioned ARS database, where the percentage of MRSA among *S.aureus *strains is provided for a large number of hospitals over time. According to this database a further increase of the percentage of MRSA was observed between 2008 and 2010 https://ars.rki.de.

Our data lead to the conclusion that a unit based surveillance approach (at least in the ICUs) is more useful for reducing nosocomial (i.e.acquired) MRSA rates compared to a hospital based approach. From the hospital level, the infection control staff has to identify the most problematic units and to provide a feedback on the unit level in order to stimulate appropriate interventions on the unit level.

## Abbreviations

MRSA: Methicillin resistant Staphylococcus aureus; KISS: German national nosocomial infection surveillance system; ICU: Intensive care unit; MDRO: Multi drug resistant organisms; IRR: Incidence rate ratio; ARS: Antibiotika Resistenz-Surveillance.

## Competing interests

The authors declare that they have no competing interests.

## Authors' contributions

PG had the idea for this analysis, drafted and finalized the paper. FS performed the statistical analysis, IC help with data retrieval and contributed to the content. CG helped with data retrieval and supervised writing of the paper. All authors read and approved the final manuscript.
